# Dynamic rewiring of biological activity across genotype and lineage revealed by context-dependent functional interactions

**DOI:** 10.1186/s13059-022-02712-z

**Published:** 2022-06-29

**Authors:** Eiru Kim, Lance C. Novak, Chenchu Lin, Medina Colic, Lori L. Bertolet, Veronica Gheorghe, Christopher A. Bristow, Traver Hart

**Affiliations:** 1 Department of Bioinformatics and Computational Biology, The University of Texas MD Anderson Cancer Center, Houston, TX 77030 USA; 2 Present Address: Novartis Institutes for BioMedical Research (NIBR), San Diego, CA USA; 3 TRACTION, The University of Texas MD Anderson Cancer Center, Houston, TX 77030 USA; 4 UTHealth Graduate School of Biomedical Sciences, The University of Texas MD Anderson Cancer Center, Houston, TX USA; 5 Department of Cancer Biology, The University of Texas MD Anderson Cancer Center, Houston, TX 77030 USA

## Abstract

**Background:**

Coessentiality networks derived from CRISPR screens in cell lines provide a powerful framework for identifying functional modules in the cell and for inferring the roles of uncharacterized genes. However, these networks integrate signal across all underlying data and can mask strong interactions that occur in only a subset of the cell lines analyzed.

**Results:**

Here, we decipher dynamic functional interactions by identifying significant cellular contexts, primarily by oncogenic mutation, lineage, and tumor type, and discovering coessentiality relationships that depend on these contexts. We recapitulate well-known gene-context interactions such as oncogene-mutation, paralog buffering, and tissue-specific essential genes, show how mutation rewires known signal transduction pathways, including RAS/RAF and *IGF1R*-*PIK3CA*, and illustrate the implications for drug targeting. We further demonstrate how context-dependent functional interactions can elucidate lineage-specific gene function, as illustrated by the maturation of proreceptors *IGF1R* and *MET* by proteases *FURIN* and *CPD*.

**Conclusions:**

This approach advances our understanding of context-dependent interactions and how they can be gleaned from these data. We provide an online resource to explore these context-dependent interactions at diffnet.hart-lab.org.

**Supplementary Information:**

The online version contains supplementary material available at 10.1186/s13059-022-02712-z.

## Background

Development of genome-wide CRISPR screening half a decade ago facilitated robust determination of genes required for in vitro proliferation across cancer types [1–3]. Ongoing efforts to identify tumor-specific vulnerabilities encompass hundreds of cancer cell lines [4–7] across dozens of tissue types. Previously, we and others demonstrated that functionally coherent modules of genes can be extracted from CRIPSR screens by measuring the similarity of gene knockout fitness profiles across all gene pairs, generating a functional interaction network based on coessentiality [8–13]. Many such modules found in the network encode well-annotated biological processes, enabling robust functional prediction for uncharacterized genes [14–16]. Some modules, however, are associated with specific tissues or genomic perturbations, such as the *ESR1*-*FOXA1* pathway in ER+ breast cancer cell lines and the *BRAF*^*V600E*^ module in melanoma cells [9, 17, 18]. In this latter cluster, gene mutation is so conflated with tissue specificity that b-RAF appears tightly linked with melanocyte-specific transcription factors *MITF* and *SOX10* but does not show significant correlation with its known protein interaction partner c-RAF (encoded by the *RAF1* gene).

We hypothesized that mutations in specific genes, and more broadly the genetic and epigenetic state of cells, could rewire these coessentiality networks and that disentangling this rewiring could elucidate the biological and therapeutic implications of genetic lesions and tissue-specific diseases. Context-specific interaction rewiring or differential network analysis is a useful tool for understanding how fixed genomes can emerge into heterogeneous cellular and tissue morphologies and phenotypes [19–21]. In prior studies, comparison of coexpression between two contexts selected from a pool were introduced to discover context-dependent interactions [22–24]. In the pre-CRISPR era, integrative functional interaction networks were exploited to prioritize disease genes [25–27] and included efforts to identify tissue-specific networks [28, 29], but these approaches lack the power of inferred functional interaction provided by coessentiality networks derived from screens in human cells. Recent examinations of coessentiality networks such as CEN-tools [18] and FIREWORKS [17] offer the ability to browse how correlates of a query gene vary by background, but offer little ability to interpret the results. Moreover, the ability to go from lesion of interest to lesion-associated network rewiring is limited.

In this study, we developed a framework of identifying dynamic relationships in cancer dependency data associated with differing functional contexts such as variations in tissue of origin and/or genomic lesions. We first categorized genomic perturbations using molecular profiles from the Cancer Cell Line Encyclopedia. Then, we investigated which genomic features were associated with synthetic gene essentiality by applying a logistic regression model, and used these features to search for associated network rewiring. Rewired networks can carry information on the efficacy of targeted drugs, the biological impact of specific lesions, and the tissue-specific activity of genes.

## Results

### Associating cellular context with emergent gene essentiality

Understanding the causal, or at least associative, basis of variation in gene essentiality is important in matching the right anticancer drugs with responsive tumors [30]. For example, *KRAS* is essential when it undergoes oncogenic mutation [31–33], and SWI/SNF complex member *ARID1B* is essential where its paralog *ARID1A* is deleted [34]. To globally decipher these essential gene/context relationships, we first systematically categorized genomic lesions into gain of function/hotspot (GOF) or loss of function (LOF) using mutation data from 808 cell lines in the Cancer Cell Line Encyclopedia [35] that have matching CRISPR screen data from the Cancer Dependency Map [5–7]. We added cell line metadata including lineage/tumor type, as well as a feature describing EMT state based on the ratio of *CDH1* to *VIM* expression (see “Methods”). After de-duplication and filtering, we retained 2918 binary features as predictor variables.

**Fig. 1:**
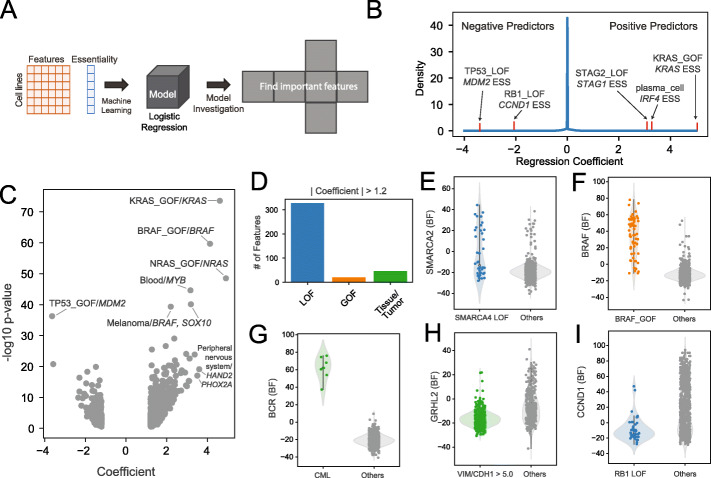
Underlying drivers of gene essentiality. **A** Schematic of the process for finding important contexts for a gene. We input binary feature vectors across all cell lines and a vector of essentiality of a gene and trained a logistic regression model. Then, we investigated coefficients of features to determine the features important for predicting gene essentiality. **B** Distribution of regression coefficients collected from prediction models of all genes. Positive coefficients reflect gene essentiality in the presence of the feature, and negative coefficients reflect gene essentiality in the absence of the feature. **C** Volcano plot demonstrates consistency between *P*-values of Fisher exact test (Y axis) and regression coefficients (X axis). **D** Category of 393 features with |regression coefficient| > 1.2. **E–I** Examples of predictions from **E** paralog interaction, **F** oncogene addiction, **G** tissue-specific essential genes, **H** EMT expression signature, and **I** negative association. BF, Bayes Factor from BAGEL2 analysis of DepMap data (positive = essential, negative = nonessential)

We used a machine learning approach to estimate the effect of genomic perturbations on variably essential genes (Fig. 1A). We chose logistic regression with an elastic net penalty because it handles binary predictors, because gene essentiality is readily binarized for use as a response variable, and because the L1 component of the elastic net forces most coefficients to zero, resulting in a model that is easily interpreted. We used a binary measure of gene essentiality because we judged that predicting whether a gene is or is not essential in a given background/cell line is more relevant than predicting variation in a continuous measure of fitness. Moreover, the log Bayes Factor output from the BAGEL2 algorithm [36], our chosen analysis pipeline, combines effect size (fitness score) and statistical significance into a single metric and, though a powerful classifier of essential and nonessential genes, is not amenable to cross-sample comparisons without normalization [5, 9]. For each gene, we generated a binary essentiality vector (defining essential as BF ≥10; see “Methods”) across 808 good quality cell lines in DepMap 20Q4 data for use as a response variable, then filtered for those genes that are essential in at least 1% but not more than 80% of the 808 cell lines, resulting in *n* = 2987 response genes.

We then generated a matrix of genetic and metadata features for each cell line, for use as a feature table of predictor variables. For each of the 808 cell lines, each gene (*n* = 18,111) was classified as loss of function (LOF) or gain of function (GOF) based on mutation calls from the Cancer Cell Line Encyclopedia. Epithelial vs. mesenchymal state was determined by CDH1/VIM mRNA expression ratio. Other cell line metadata, including unique labels associated with lineage/disease type, and cell culture conditions, and patient sex, were each transformed into binary feature vectors. All features (*n* = 36,406) were then filtered, removing those describing six or fewer cell lines (*n* = 33,475) and de-duplicating highly correlated features to prevent collinearity. The final de-duplicated binary feature set comprised 2918 predictor variables for the regression model (see “Methods” for details).

We then used logistic regression with the same table of 2918 features to predict the essentiality profile of each of the 2987 response genes independently. This approach is similar to that used by Lord et al. [37], but our logistic regression is a binary classifier of gene essentiality rather than their linear regression on continuous fitness effects. After calculating all regression models, we investigated which genomic features significantly contributed to predicting essentiality (Fig. 1B, C). We collected coefficients of genomic features across all prediction models of genes, which loosely represent the magnitude of predictive power. A positive coefficient of a genomic feature indicates that the genetic lesion is associated with the gene dependency (e.g., KRAS_GOF/*KRAS* essentiality), and a negative coefficient indicates that gene essentiality is associated with the absence of the lesion (e.g., TP53_LOF/*MDM2* essentiality). Coefficients showed a similar trend with *P*-values derived by the Fisher exact test between a vector of genomic lesions and a vector of gene dependency across cell lines (Fig. 1C). By considering strong coefficients (see “Methods” and Additional file 1: Figure S1), we identify 393 genomic features strongly predictive of variation in gene essentiality, including 327 loss of function mutations, 20 gain of function mutations, and 46 features derived from cell metadata (Fig. 1D). We recapitulated positive associations in paralog buffering (e.g., SMARCA4_LOF/*SMARCA2*^*ess*^, Fig. 1E), oncogenic mutation-induced essentiality (e.g., BRAF_GOF/*BRAF*^*ess*^, Fig. 1F), and tumor-specific dependencies (e.g., CML/*BCR*^*ess*^, Fig. 1G), as well as negative associations such as epithelial transcription factor *GRHL2* essentiality in cells with low *CDH1/VIM* expression ratio (Fig. 1H) and increased essentiality of cyclin D1 (*CCND1*) in cells with wildtype *RB1* (Fig. 1I). These results are highly concordant with previous predictive models (Additional file 1: Figure S1) [5, 37] and known biology and provide a meaningful set of features for exploring context-dependent functional interaction rewiring. The table of all coefficients is included as Additional file 2.

### Measuring context-dependent network rewiring

With the identification of contexts associated with variation in gene essentiality, we expanded our view to functional interaction rewiring associated with genomic and lineage features. In general, genes with correlated fitness profiles across a diverse panel of cell lines tend to operate in the same biological processes, such as enzymatic or signal transduction pathways, often as subunits in the same protein complex. While this correlation provides a powerful framework for inferring gene function and the modular structure of the cell [8–13], it relies on covariation in the underlying gene essentiality vectors but offers no insight into the source of this variation. We hypothesized that, by systematically removing rationally selected subsets of cell lines and measuring the effect on pairwise correlation, we could identify the drivers of this covariation and, in turn, infer the causal basis for the emergent essentiality of biological processes.

**Fig. 2:**
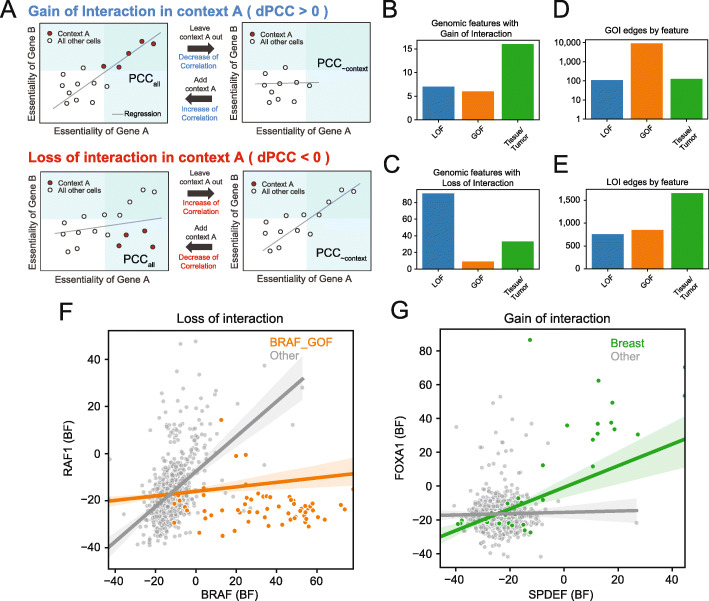
A framework to identify dynamics in coessentiality network. **A** Classes of context-dependent interaction. In all cases, a differential Pearson correlation coefficient (dPCC) for each gene pair is calculated as the difference between the global network (PCC_all_) and the PCC from all cells except those harboring the feature of interest (PCC_~context_). **B**, **C** The number of features associated with gain or loss of interaction. **D**, **E** the number of gained or lost interactions, by feature type. Nearly all GOI edges are associated with TP53_GOF mutations. **F**, **G** Examples of context-dependent loss and gain of interaction. Axes are Bayes Factors of the genes

Although differential coessentiality offers a seemingly straightforward way to approach context rewiring of functional interaction networks, the method is beset by complications. One is that, for many genes, physiologically relevant knockout fitness defects are typically only seen in a few cell lines. A related issue is that there are frequently too few cell lines associated with a given context for correlation to be an accurate predictor of functional interaction within the context; e.g., a CML-only coessentiality network has limited meaning if there are only seven CML cell lines. To overcome these difficulties, we designed a strategy comprising a leave-one-out test and bootstrapped network comparison analysis to investigate snapshots of interaction rewiring in essentiality data associated with our features of interest. In the leave-one-out test, interaction rewiring was measured as a differential Pearson correlation coefficient (dPCC) of a context by taking the PCC of knockout fitness profiles (in our case, quantile normalized logBF scores from BAGEL2) from all cell lines (PCC_all_) and subtracting the PCC using all cell lines except those carrying the feature of interest (PCC_~context_) (Fig. 2A). We classified a positive dPCC as a gain of interaction (GOI) because the PCC depends on the presence of cells with the feature of interest (Fig. 2A, top). Conversely, we describe a negative dPCC as a loss of interaction (LOI) because the background PCC is improved by removing cells with the feature of interest (Fig. 2A, bottom).

We measured dPCC for the 393 features identified in logistic regression models as significant predictors of gene essentiality, and measured significance by bootstrap resampling to estimate a null distribution of dPCC (see “Methods”). We identified 30 features significantly associated (*P* ≤0.001) with gain of interaction (Fig. 2B) and 133 features, including 91 LOF mutants, associated with loss of functional interaction (Fig. 2C) in the cell lines studied. These features are associated with 9227 gain of interaction and 3261 loss of interaction events (Fig. 2D,E). Notably, of the gain of interaction events, 97% (*n* = 8958) are associated with TP53 gain of function/hotspot mutations, leaving only 269 interactions associated with the remaining 29 features. A list of top dPCC hits is included as Additional file 3.

Two case studies illustrate the effect of context-dependent rewiring of functional interaction networks. RAS and RAF kinases are core members of RTK signaling pathways for proliferation and differentiation [38–42]. Within the canonical MAP kinase signal transduction pathway, b-RAF (*BRAF*) and c-RAF (*RAF1*) form a heterodimer to transmit phosphorylation signals from upstream RAS genes to downstream MEK/ERK targets. In *BRAF*-driven cancers, oncogenic mutation constitutively activates *BRAF* and obviates the need for c-RAF binding. In the global coessentiality network, *BRAF*^*V600E*^ and *BRAF*^*wt*^ cell lines are mixed, diluting the ability to discover the wildtype *BRAF*-*RAF1* relationship (background PCC = 0.124). Removing *BRAF*^*V600E*^ cell lines boosts this correlation to 0.478 (dPCC = −0.363; *P* < 0.001, see “Methods” for details of statistical tests); thus, we infer that BRAF_GOF mutation is causal for a loss of interaction between *BRAF* and *RAF1* (Fig. 2F). Similarly, transcription factor *FOXA1* is essential in a subset of lung and breast cancers but shows a breast-specific functional interaction with ETS family transcription factor *SPDEF* (Fig. 2G).

### Reconstruction of directionality in signaling pathways

The dynamic rewiring of functional interactions resulting from cancer-associated mutation has predictable consequences. As previously observed, mutation of the *TP53* tumor suppressor removes the cell’s reliance on *MDM2* and related genes to suppress the proapoptotic activity of wildtype p53 protein. Similarly, constitutive activation of signaling proteins by oncogenic mutation activates downstream signaling partners while removing the need for upstream activation signals. Here we describe two such pathways.

#### RAS/RAF signaling pathway

**Fig. 3:**
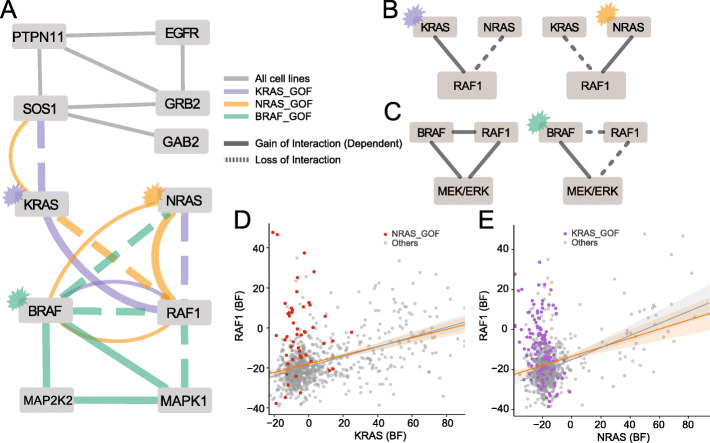
Interaction rewiring in RAS/RAF pathway. **A** A network diagram of differential interactions in the RTK/MAP kinase signaling cascade caused by mutations on KRAS, NRAS, and BRAF. **B** KRAS gain of function mutant caused loss of NRAS-RAF1 interaction and NRAS mutant caused loss of KRAS-RAF interaction. **C** RAF1 is required for transduction signal to MEK/ERK signal when BRAF is wildtype, while BRAF mutant does not require RAF1 and led to loss of RAF1 interaction with BRAF and the downstream pathway. **D**, **E** Scatter plots of interactions between RAF1 and two RAS proteins

Mutations in the RAS pathway are major drivers of numerous cancers. RAS family members *KRAS* and *NRAS* are frequently mutated in colorectal and pancreas adenocarcinoma, and multiple myeloma and melanoma, respectively. *BRAF* is a major driver gene of melanoma and thyroid adenocarcinoma [43, 44]. Oncogenic GOF lesions in these genes caused significant intra- and inter-pathway interaction rewiring (Fig. 3A). *KRAS* and *NRAS* are usually nonessential in CRISPR data; however, mutation or amplification of these genes induces hyperactivation and strong mutually exclusive essentiality. We found that interaction rewiring of GOF mutations of *KRAS*, *NRAS*, and *BRAF* induced gain of interaction with downstream genes and disconnected the network from upstream genes through loss of interaction (Fig. 3A). For example, *KRAS* mutation amplifies the link with downstream signaling partner *RAF1* (PCC_all_ = 0.390, PCC_~KRAS_GOF_ = 0.239, dPCC = 0.151, *P* < 0.001), severs the link between *RAF1* and *KRAS* paralog *NRAS* (PCC_all_ = 0.305, PCC_~KRAS_GOF_ = 0.472, dPCC = −0.167, *P* < 0.001), and disconnects MAP kinase signaling from the upstream *EGFR* receptor tyrosine kinase signal transduction module (*KRAS-SOS1* PCC_all_ = −0.012, PCC_~KRAS_GOF_ = 0.184, dPCC = −0.197, *P* < 0.001). Similar behavior is seen in *NRAS* and *BRAF* GOF cell lines (Fig. 3B–E). Additional rewiring by *BRAF* mutation abrogates interaction with dimerization partner *RAF1*, consistent with the known biology of *BRAF* mutation. We found that *BRAF* mutation induced loss of interaction between *BRAF* and *RAF1* while *BRAF* and *RAF1* are well correlated in wildtype *BRAF* cell lines (Fig. 3A, C).

#### IGF1R/PIK3CA signaling pathway

**Fig. 4:**
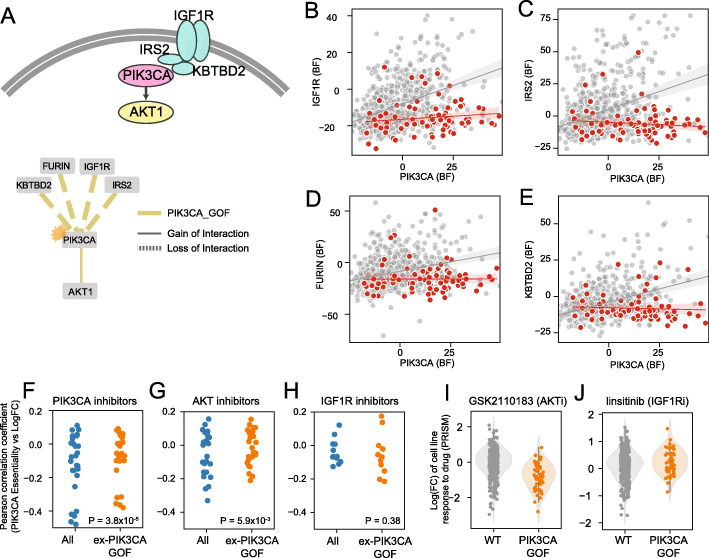
Dynamics of network rewiring on IGF1R-PIK3CA pathway. **A** A visualization of IGF1R-PIK3CA pathways (upper panel) and a differential network of pathway genes caused by PIK3CA GOF mutation. Upstream pathway genes were disrupted by mutation while correlation with downstream pathway gene AKT1 was boosted. **B–E** Scatter plots of interactions of PIK3CA upstream genes **B** IGF1R and **C)** IRS2, plus **D** IGF1R maturation factor FURIN, and **E** KBTBD2. **F–H** Comparisons of effectiveness of **F** PIK3CA inhibitors, **G** AKT inhibitors, and **H** IGF1R inhibitors in all cells (blue) and cells excluding PIK3CA GOF mutations (orange), from PRISM. **I** AKT inhibitor is more effective in PIK3CA GOF, while **J** IGF1R inhibitor is more effective in PIK3CA wt

The *PIK3CA* gene encodes a protein kinase that mediates signaling from insulin-like growth factor receptor *IGF1R* to the *AKT* pathway [45] (Fig. 4A), is frequently mutated in a number of cancers, and is itself the target of several chemotherapeutic agents. When mutated, *PIK3CA* is hyperactivated and induces downstream pathways without reliance on *IGF1R* signals [46]. In our differential network analysis, *PIK3CA* gain of function mutation causes not only loss of interaction with *IGF1R* receptor (Fig. 4B) but also the insulin receptor substrate gene *IRS2*, which encodes a protein involved in *IGF1R* signal transduction (Fig. 4C). In addition, PIK3CA_GOF causes loss of interaction with *FURIN* (Fig. 4D), a protease required for maturation of the *IGF1R* receptor [47] (*FURIN*-*IGF1R* PCC_all_ = 0.540, *n* = 808 cell lines) and uncharacterized gene *KBTBD2* (Fig. 4E). The high *KBTBD2*-*IRS2* background correlation (PCC_all_ = 0.483), and the loss of interaction with *PIK3CA* upon oncogenic mutation (PCC_all_ = 0.293, PCC_~PIK3CA_GOF_ = 0.400, dPCC = −0.107, *P* < 0.001), support a functional interaction between *KBTBD2* and *IRS2*, possibly involving protein maturation or signaling.

We further investigated the impact of interaction rewiring on drug efficacy. We compared the effect of drugs targeting *PIK3CA*, *AKT1*, and *IGF1R*, between wildtype and mutant *PIK3CA* cell lines in PRISM [48] 19Q3 data (Fig. 4F–H), where strong drug efficacy is shown as negative log fold change and negative correlation between BF profiles from CRISPR screens and drug response profiles from PRISM indicates concordance. Cell lines harboring a *PIK3CA* oncogenic mutation were significantly more sensitive to AKT inhibitor GSK2110183 (Fig. 4I). Conversely, *IGF1R* inhibitor linisitib is effective only in a subset of cell lines with wildtype *PIK3CA* alleles (Fig. 4J). Together, these findings are consistent with the mutational rewiring of the functional interaction network and demonstrate that context-dependent networks can inform drug sensitivity.

### Tissue-specific functional interaction

**Fig. 5:**
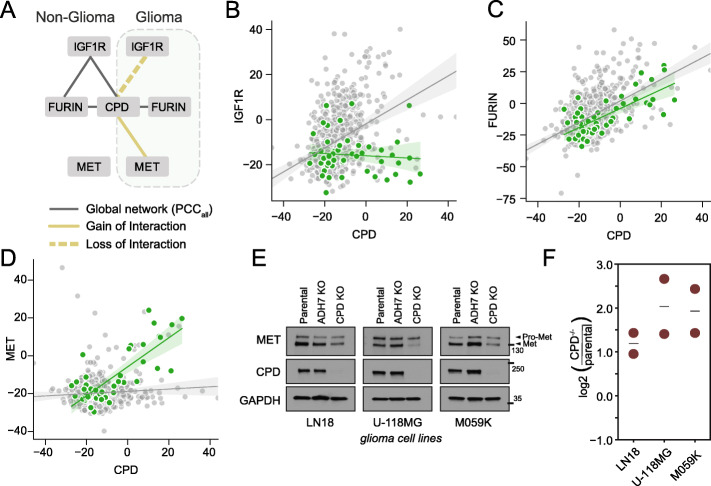
Tissue-specific network rewiring. **A** A network diagram of interaction rewiring of CPD in glioma cell lines. **B** IGF1R-CPD loss of interaction was detected in glioma cell lines. **C** CPD-FURIN interaction was correlated regardless of tissue type. **D** CPD is correlated with MET instead of IGF1R in glioma cell lines. **E** Abundance of proMET and mature MET receptor in three glioma cell lines, in response to CPD knockout or negative control (ADH7ko). **F** Densitometry of Western blots shows ratio of proMET to MET more than doubles in CPD^−/−^ cells

While *IGF1R* is associated with its canonical downstream signaling partner *PIK3CA*, its strongest association in the global coessentiality network is with *FURIN* (PCC_all_ = 0.540), reflecting the role of *FURIN* in *IGF1R* receptor maturation [47]. *FURIN* is also strongly associated with carboxypeptidase D (*CPD*), recently reported to be required for pro-*IGF1R* maturation in lung adenocarcinoma [49] (Fig. 5A). Interestingly, the link between *CPD* and *IGF1R* is largely abrogated in glioma cells (Fig. 5B), while the *CPD*-*FURIN* relationship is maintained (Fig. 5C).

Together these observations suggest that the *CPD*-*FURIN* activity is required for maturation of a different protein in glioma cells. Notably, hepatocyte growth factor receptor tyrosine kinase *MET* shows a strong gain of interaction with *CPD* in glioma (Fig. 5D). *MET* polypeptide, like *IGF1R*, is proteolytically processed into a mature receptor through cleavage into alpha and beta subunits and linkage via disulfide bonds [50]. Our context-dependent coessentiality network predicts functional linkage between *CPD* and *MET*, specifically in glioma cells, and prior knowledge of *CPD* function elsewhere suggests *CPD* may be involved in posttranslational processing of *MET* proreceptor, a hypothesis also suggested by Han et al. [49].

To test this prediction, we used a dual-guide CRISPR/enCas12a vector (see “Methods”) to knock out either *CPD* or negative control *ADH7* in glioblastoma cell lines and examined *MET* protein. We selected three brain cancer cell lines from PICKLES [51] where the *MET* receptor was expressed but not essential (LN18, U118MG, and M095K). As measured by Western blotting, under normal conditions, MET is more abundant than its precursor proMET, indicating efficient posttranslational maturation of the polypeptide (Fig. 5E). In contrast, in *CPD*^−/−^ cells, the ratio of proMET to MET is, on average, 3.6 times that of parental cells (Fig. 5F; Additional file 4). These observations are consistent with *CPD* playing an important role in maturation of the *MET* proreceptor in glioblastoma cells.

### Diffnet: explore dynamic functional interactions

A website is available at https://diffnet.hart-lab.org for interactive visualization of context-dependent functional interactions. Users can browse or search predefined sets of genetic lesions or tissue/tumor types and see the networks that are associated with differential coessentiality in that context as well as a table of network edges along with key parameters, including PCC, dPCC, and empirical *P*-values. Clicking any edge in the network view will display a scatterplot of logBF scores for the two connected genes across all cell lines in the DepMap data, annotated by whether they are in the selected context. A screenshot of the BRAF/RAF1 change in interaction in the BRAF_GOF context is shown in Additional file 1: Figure S2.

## Discussion

In this study, we present a framework for understanding the differential wiring of cells across genotypes and lineages. Using genetic mutation information and cell line metadata, we investigated contexts that cause emergent essentiality by analyzing coefficients obtained from a logistic regression model trained using CRISPR screen data. Using this approach, we identified numerous context-gene associations, including known interactions such as paralog buffering, oncogenes, and tissue-specific essential genes.

We then examined the rewiring of functional interaction networks in the presence of these contexts. We developed a strategy which compares the strength of interaction between two genes, as measured by the Pearson correlation coefficient of their normalized fitness vectors across all samples, to the correlation derived from samples that exclude a specified context, or a “leave-one-out” test. We measured the significance of these changes by bootstrapping a null distribution for every gene pair in every context. Rewiring detected by our approach showed concordance with biological knowledge and discovered new putative context-dependent functional interactions, demonstrating its potential for functional genomics and cancer targeting. We provide a web-based interface for exploring the network edges rewired by mutation and/or lineage at diffnet.hart-lab.org.

## Conclusions

The approach derived herein attempts to address one of the key questions arising from the use of coessentiality, or indeed any gene similarity approach, to predict gene co-functionality. These methods offer a powerful approach for predicting gene function, identifying disease genes, and reducing the search space of potential combinatorial gene effects to tractable levels. Coessentiality depends on some underlying variation in gene essentiality across the dataset; genes which are always essential or never essential rarely appear in these networks [9]. In most approaches, the source of this variation is neither known nor questioned; however, the question is of high interest because identifying the causal basis for emergent essentiality would provide direct association of biomarkers with potential cancer targets as well as insight into the critical biological processes in different cells.

Another feature of raw coessentiality networks is that, as with prior efforts to build integrative functional interaction networks [26], they represent an integration of all contexts present in the cell lines or data sets from which they are derived. Functional interactions between the same genes may differ across contexts [28, 52]; for example, the roles of *FURIN* and *CPD* in promoting the maturation of different cell surface receptors in different tissues. Combining these contexts dilutes the overall correlation between genes with strong but context-dependent functional interaction. The approach we describe here offers a path towards understanding how context-dependent interactions can be gleaned from these data.

## Methods

### Preprocessing publicly available CRISPR screens

Raw read counts of CRISPR screens used in this study were downloaded from DepMap 20Q4 (*n* = 808 cell lines). For DepMap screens, we removed guide RNAs targeting multiple protein coding genes based on the guide map obtained from the DepMap database and the CCDS protein coding gene annotation to avoid genetic interaction effects. After that, we applied the BAGEL2 pipeline [36] to measure gene essentiality information for each cell line. Gene knockout fitness phenotype is reported as a log Bayes Factor (BF), with positive scores indicating likelihood of essentiality.

### Predictive features and response variables for the logistic regression model

Genetic lesions were classified as gain or loss of function based on mutation calls described in the Cancer Cell Line Encyclopedia. For each of the 808 cell lines, each gene (*n* = 18,111) was classified as loss of function (LOF) if it carried a lesion whose “Variant_annotation” annotation was “damaging.” Genes were characterized as gain of function (GOF) if they carried a lesion where either isTCGAhotspot or isCOSMIChotspot = True and if “Variant_annotation” was “other non-conserving.” Other lesions were not classified. See notebook “pre-step01-clean_data” for details.

CCLE Gene expression data in logTPM was downloaded from the CCLE portal at the Broad (*n* = 718 cell lines with matching CRISPR data from Avana 20q4). EMT state was determined by CDH1/VIM expression log ratio (logTPM_CDH1_ − logTPM_VIM_), which resulted in a bimodal distribution across all cell lines. Each cell line was assigned CDH_VIM_hi if the log ratio > 1, or CDH_VIM_lo for log ratio < −4 (Additional file 1: Figure S3). This CDH_VIM_hi and CDH_VIM_lo were the only gene expression feature used in the classifier. See notebook “step01-merge_features” for details.

Other cell line metadata, downloaded from the CCLE portal at the Broad (*n* = 808 cell lines with matching CRISPR data from Avana 20q4), were transformed into binary features. Each unique entry for “lineage,” “lineage_subtype,” “lineage_sub_subtype,” “culture_type,” and “sex” was considered a unique feature vector. See notebook “step01-merge_features” for details.

All features were merged into one matrix (808 cell lines × 36,406 features). Features describing six or fewer cell lines (*n* = 33,475) were excluded. Remaining features were then de-duplicated by calculating an all-by-all correlation matrix, and for every pair of features with PCC ≥0.9, the smaller feature (the one describing fewer cell lines) was discarded. For features with equal size and perfect overlap (e.g., “plasma_cell” and “multiple_myeloma”), one was selected at random and discarded. The final de-duplicated binary feature set comprised 2918 predictor variables for the regression model. See notebook “step02-remove_duplicates” for details.

Gene essentiality was binarized for use as a response variable. Genes with missing values in the Bayes Factor table (*n* = 17) were removed from the dataset. Then, for each cell line, genes with BF ≥10 were classified as essential and all others nonessential, for a set of 18,094 genes in 808 cell lines. We used a binary rather than continuous response variable because, in this study, we seek to identify predictors of whether a gene does or does not have a fitness defect, not to predict variation in that defect. Moreover, while the magnitude of positive log BF may be a proxy for severity of fitness defect, the magnitude of negative log BF is usually not (though see [53] for exceptions). Bayes Factor combines statistical confidence and effect size into a single output, and the dynamic range of BF scores from each screen is highly dependent on that screen’s quality [36, 54]. Bayes Factors are not directly comparable across screens without normalization (see Global normalization, below). See notebook “step02-remove_duplicates” for details on this processing step.

#### Logistic regression model

Each gene’s binary essentiality vector was used as the response variable in a logistic regression model, using the binary features described above. Genes whose essentiality is invariant across the cell lines are uninteresting; therefore, we excluded genes essential in less than 1% or more than 80% of the samples (Additional file 1: Figure S3). The remaining set included 2987 genes.

A logistic regression model was implemented in Python using the LogisticRegression function in scikit-learn, with an elastic net penalty (L1 ratio = 0.25). Processing time took 78 min on an AMD 5950X CPU with 32 threads. The coefficients for each feature/gene prediction were combined into a single matrix. The intercept of each model closely matched the background probability of gene essentiality (Additional file 1: Figure S1b). For each of the 2987 response genes, the maximum and minimum feature coefficients were determined and, based on histograms of the maximum and minimum values (Additional file 1: Figure S1cd), |coeff| > 1.2 was chosen as a threshold for strong feature-gene association. The feature-gene coefficients matrix was flattened to a list and sorted by coefficient, and this list was used for subsequent analyses. A total of 393 unique features were predictive of gene essentiality with |coeff| > 1.2. See notebook “step03-do_regression” and “step03a-audit_regression” for details. The matrix of all feature-gene coefficients is included as Additional file 2.

### Measurement of emergent network rewiring in CRISPR dataset

#### Global normalization and correlation

To calculate the baseline coessentiality network, we first quantile normalized the log BF table of 18,094 genes with no missing data × 808 cell lines. Quantile normalization was performed without a reference distribution, setting each gene to its rank mean; e.g., the top-ranked gene in each sample was assigned a value equal to the mean BF of all top-ranked genes in 808 samples. The global Pearson correlation network of all gene pairs was then calculated using the Python numpy function corrcoef, resulting in an 18 k × 18 k matrix of Pearson correlation coefficients hereafter referred to as PCC_all_.

#### Leave-one-out test

To test for dynamic functional network rewiring, we measured the differential Pearson correlation coefficient (dPCC) between the PCC_all_ global network and the network derived from a subset of cell lines that exclude a given context (PCC_~context_). The formula of dPCC for each gene pair is therefore:

$$ dPCC={PCC}_{\mathrm{all}}-{PCC}_{\sim \mathrm{context}} $$

where PCC_~context_ is an 18 k × 18 k matrix of Pearson correlation coefficients derived from the [18 k × (808 − *n*) ] matrix of quantile normalized BFs, and *n* is the number of cell lines associated with the “context.” Positive dPCC represents a *gain of interaction (GOI)* because the higher PCC_all_ depends on the presence of cells with the feature of interest, and negative dPCC represents *loss of interaction (LOI)* because excluding cells associated with the feature of interest increases the PCC.

We evaluated the statistical significance of the observed dPCC (dPCC_obs_) for each gene pair/feature combination empirically. Briefly, we determined *n*, the number of cell lines associated with the feature. Then we randomly removed *n* cell lines from the set of all 808 cell lines and calculated the all-by-all PCC matrix for all pairs of genes, PCC_rand_. For each gene pair, we measured dPCC_rand_ = PCC_all_ − PCC_rand_. We repeated this process 1000× and compared the observed dPCC_obs_ to the bootstrapped distribution of dPCC_rand_ to calculate a two-sided empirical *P*-value that dPCC_obs_ is higher or lower than random expectation. In general, our sampled null distributions of dPCC_rand_ are not well estimated by classical distributions, and our estimate of *P*-value is therefore limited to a discrete distribution in steps of 0.001. We repeated this bootstrap resampling to generate null distributions of dPCC_rand_ for every gene pair for each of the 393 contexts identified by the logistic regression model (a computationally expensive process). See “calc_diff_coess_pval.py” for code and details.

It is important to note that this empirical approach does not rely the robust measurement of Pearson correlation—in fact, the approach described here exploits the Pearson’s lack of robustness in the presence of outliers. When the outliers are functionally coherent in some aspect (e.g., breast cancer cells in Fig. 2G, glioma cells in Fig. 5D), their removal in the leave-one-out test and the resulting major change in PCC is precisely the result of the Pearson’s sensitivity to outliers, and would likely not be detectable with nonparametric alternatives such as Spearman’s rank correlation that are designed to be insensitive to such changes. Of further importance is that we do not evaluate the statistical significance of any Pearson correlation, in large part because of these concerns. Our only test statistic is dPCC, measured against null distributions that we sample empirically.

### CPD knockout in glioblastoma cells

#### gRNA plasmid construction

Two gRNAs targeting ADH7 and CPD were designed by CRISPick [55]. Oligonucleotides containing two enCas12a crRNAs (crRNA-DR1-crRNA) (crRNA sequences were listed in Table 1; DR1 is drawn from [56]) were synthesized by Integrated Device Technology and cloned to the pRDA_550 one-component enCas12a/crRNA expression vector (gifted from John Doench) by BsmBI-v2 Golden Gate Assembly (New England Biolabs, E1602S) following the manufacturer’s instructions.

**Table 1:** Sequences of crRNAs

Genes	crRNA1	crRNA2
CPD	5’-CCAAGGAATAAAGCCGGGTAATG-3’	5’-GGAACAGAATCGAAGATCACTAA-3’
ADH7	5’-GCCACAGGAATCTGTCGCACAGA-3’	5’-TCTCCACAGGTCAAACCTGGTTC-3’

#### Cell culture

M059K (CRL-2365), LN-18 (CRL-2610), and U-118 MG (HTB-15) were sourced from the American Type Culture Collection (ATCC). Cell line identities were confirmed by STR fingerprinting by M.D. Anderson Cancer Center’s Cytogenetic and Cell Authentication Core (Promega Powerplex 16 High Sensitivity Assay). All cell lines were routinely tested for mycoplasma contamination using cells cultured in non-antibiotic medium (PlasmoTest Mycoplasma Detection Assay, InvivoGen).

All cell lines were grown at 37 °C in humidified incubators at 5.0% CO_2_. Cell lines were cultured in HEPES modified Dulbecco’s modified Eagle’s medium (Sigma D6171) supplemented with 10% FBS (Sigma), 1 mM sodium pyruvate (Gibco), 2 mM L-alanyl-L-glutamine dipeptide (Gibco), 1× penicillin-streptomycin (Gibco), and 100 μg/mL Normocin (InvivoGen).

Lentivirus was produced by the University of Michigan Vector Core. Virus stocks were not titered in advance. All transductions were performed in a 6-well plate format across multiple plates with a range of virus volumes and 8 μg/mL polybrene (EMD Millipore). The pool with the most optimal transduction efficiency was expanded and collected for western blotting.

Non-transduced cells were eliminated via selection with 2 μg/mL puromycin dihydrochloride (Gibco). Selection was maintained until all non-transduced control cells reached 0% viability. Once selection with puromycin was complete, three replicates were seeded in a 6-well plate format using the following amounts of viable cells: 4 × 10^5^ LN-18 per well, 6 × 10^5^ U-118 MG per well, 6 × 10^5^ M059K per well.

#### Western blots

Cells were washed with PBS and harvested in RIPA lysis buffer (Thermo Fisher Scientific, 89900) supplemented with Halt Protease Inhibitor Cocktail (Thermo Fisher Scientific, 87786). Twenty micrograms of cell lysate was denatured in Laemmli SDS-Sample Buffer (Boston BioProducts, BP-110R) and loaded on a precast 4–15% Mini-Protean TGX Stain-Free gel (Bio-Rad). After electrophoresis and transfer, the PVDF membrane (Bio-Rad) was blocked in 5% nonfat milk with TBST (Bio-Rad) at room temperature for 1 h and incubated with primary antibody overnight. MET (3127, 1:1000) antibody was purchased from cell signaling technology. CPD (A305-514A-M, 1:1000) and GAPDH (MA5-15738, 1:5000) antibodies were purchased from Thermo Fisher Scientific. The membranes were then washed in TBST three times, followed by incubation with goat anti-rabbit/Mouse secondary antibodies (Cell Signaling Technology, 1:2000–1:5000) for 1 h at room temperature. After washing with TBST, the membranes were developed by films. All western blots were repeated twice. Image quantification was performed in ImageJ.

## Supplementary Information

**Additional file 1: Figure S1.** Regression model. **Figure S2.** Screenshot of Diffnet web service at https://diffnet.hart-lab.org. **Figure S3.** Selected regression model features. **Figure S4.** Raw Western blots from Fig. 5 in main text.

**Additional file 2.** Tab-delimited table of regression coefficients, with 2918 genomic and lineage features used to predict background-specific essentiality of 2987 genes.

**Additional file 3.** Tab-delimited table of top 2644 hits from differential PCC analysis.

**Additional file 4.** Quantitative data from Western blot experiments.

**Additional file 5.** Review history.

## Data Availability

Code for the data analysis pipeline and for the *diffnet* website can be accessed on demand at the repository at Github [57] and Zenodo [58]. The diffnet website is available at https://diffnet.hart-lab.org/.
