# Supplementary material for: Dynamic rewiring of biological activity across genotype and lineage revealed by context-dependent functional interactions
Source: Genome Biol. 2022 Jun 29;23:140. doi: 10.1186/s13059-022-02712-z (PMC9241233; doi:10.1186/s13059-022-02712-z)
Supplement: Supplementary file 1 — Additional file 1: Figure S1. Regression model. Figure S2. Screenshot of Diffnet web service at https://diffnet.hart-lab.org. Figure S3. Selected regression model features. Figure S4. Raw Western blots from Fig. 5 in main text. [file 13059_2022_2712_MOESM1_ESM.docx]

**Supplementary Figures and Legends**


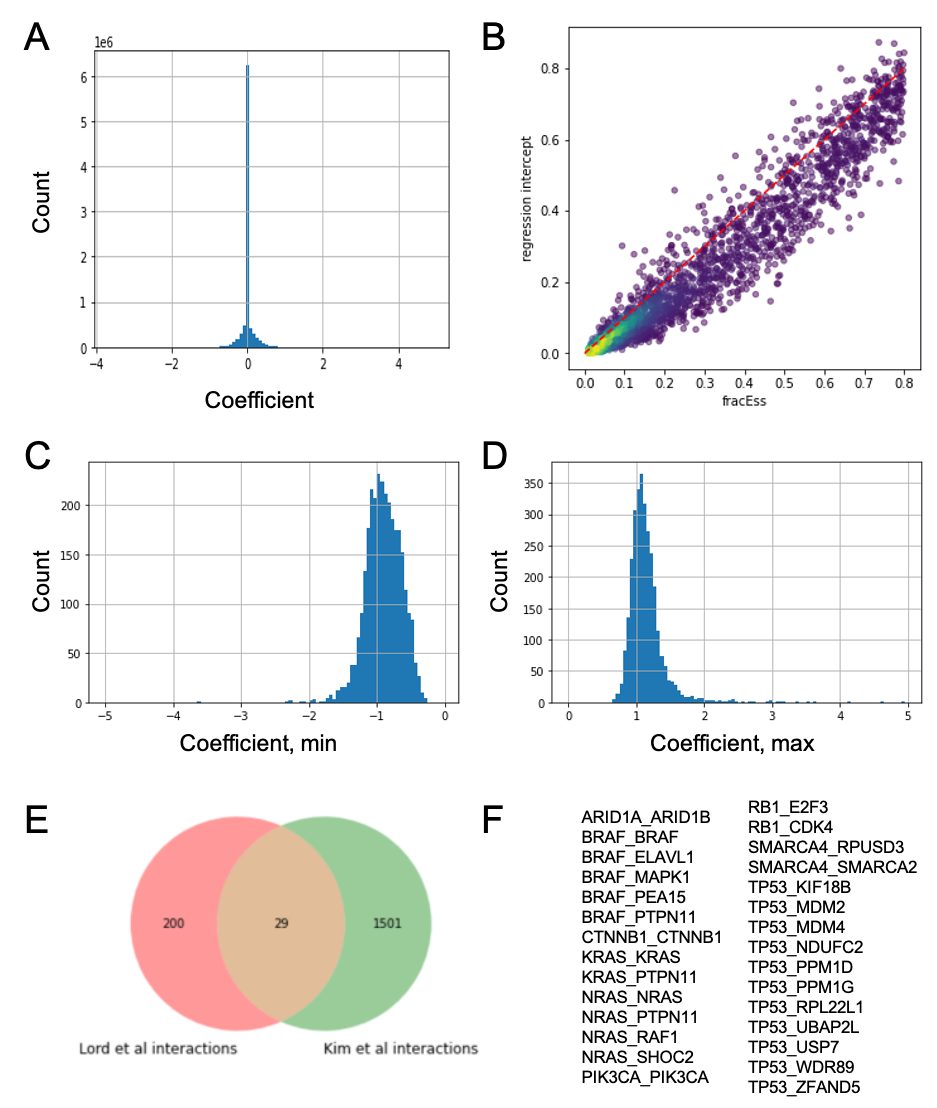


**Figure S1. Regression model.** (A) Histogram of all ~8.7 million genomic feature-response gene coefficients. Elastic net penalty forces most coefficients to zero. (B) Regression intercept (y-axis, exp(Intercept) / exp(Intercept) + 1 ) approximates frequency of gene essentiality (x-axis) across cell lines. (C, D) Distribution of minimum (C) and maximum (D) value of regression coefficient for each of 2,918 predictor variables across 2,987 predictions, from which we chose |coeff| > 1.2 for further analysis. (E) Comparison of significant feature-gene interactions between Lord et al and this study. This study includes tissue/lineage as a predictor, resulting in more interactions. (F) Interactions (in *mutated gene_response gene* format) in the Lord/Kim intersection from (E).


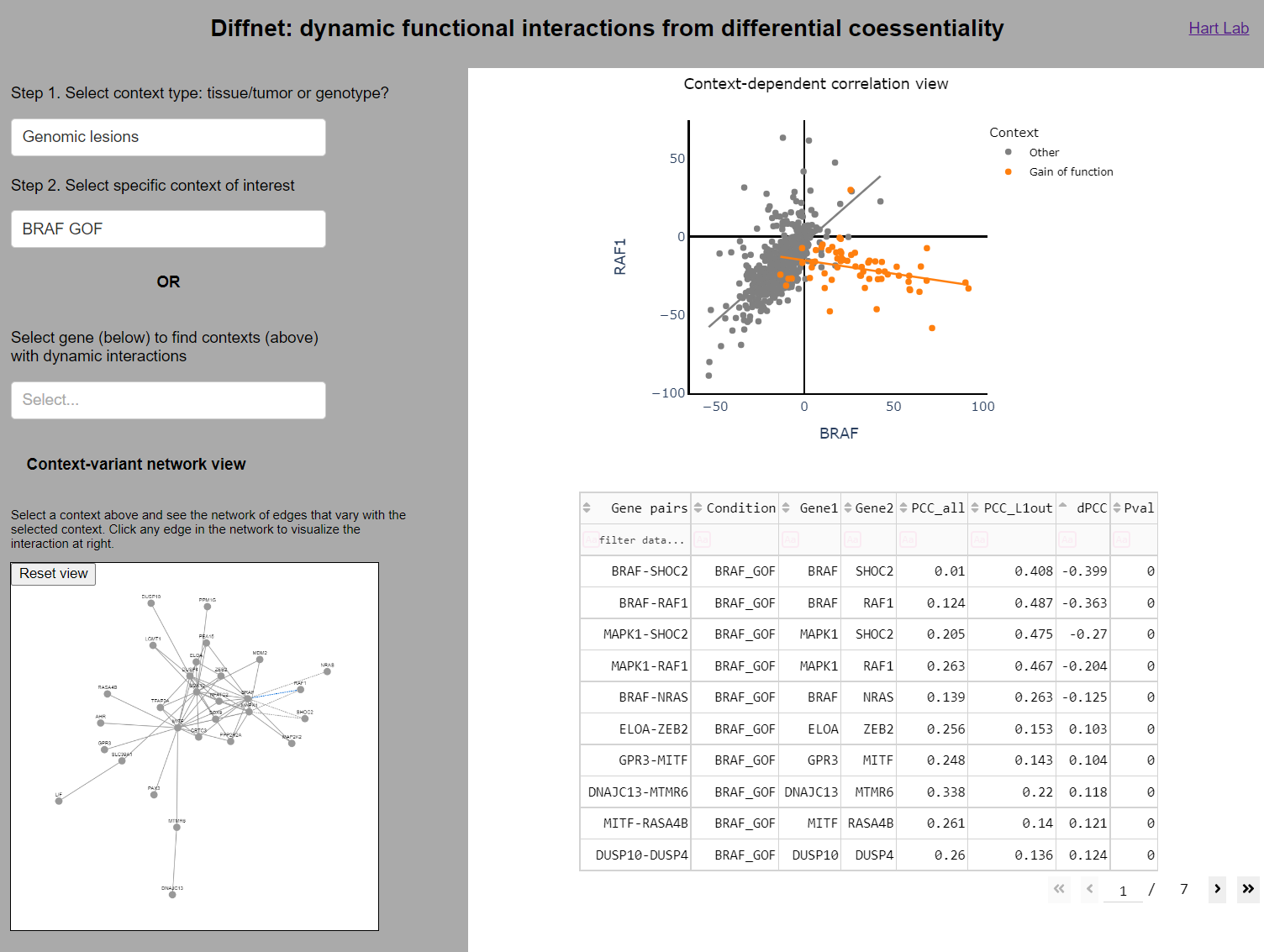


**Figure S2**. **Screenshot of Diffnet** web service at https://diffnet.hart-lab.org.


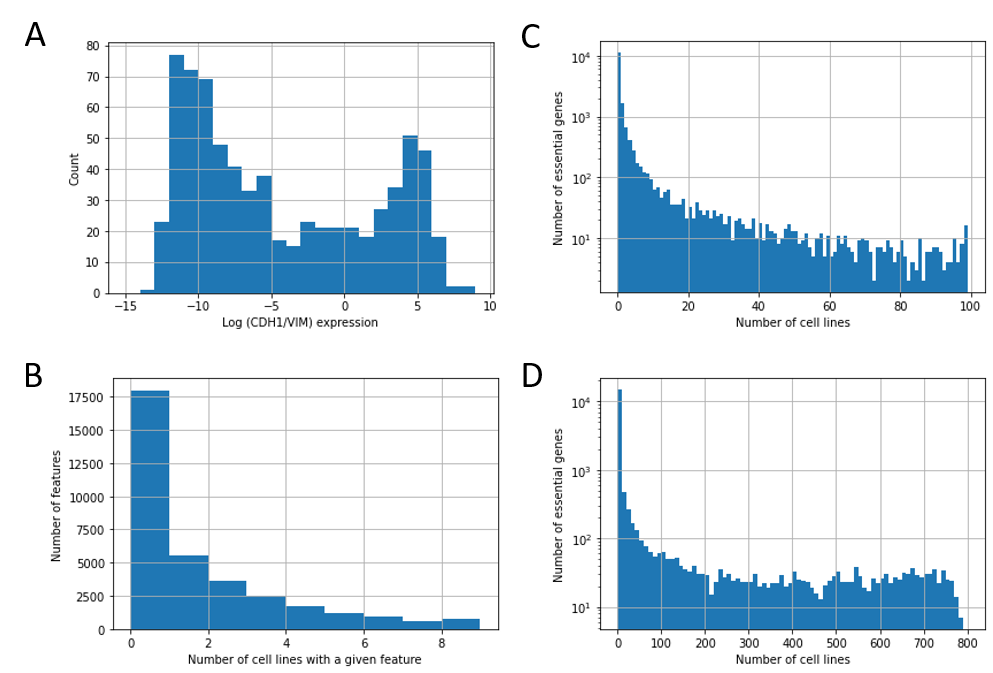


**Figure S3. Selected regression model features**. (A) Distribution of CDH1/VIM gene expression ratio. X-axis, log ratio of gene expression. Y-axis, number of cell lines (n=718 total). Cell lines with ratio > 1 were classified as CDH_VIM_hi and ratio < -4 as CDH_VIM_lo. Other cells were not labeled. (B) For each feature, the total number of cell lines labeled with that feature was measured. Most features apply to only a very small number of cells. Features present in only six or fewer cell lines were discarded. (C-D) Number of cell lines in which a gene is classified as essential, with one cell line per bin (C) or 10 cell lines/bin (D). Most genes are essential in only a few cell lines, characteristic of false positives. Cells essential in less than 1% (8 cell lines) or more than 80% (646 cell lines) of samples were discarded as invariant.

A B




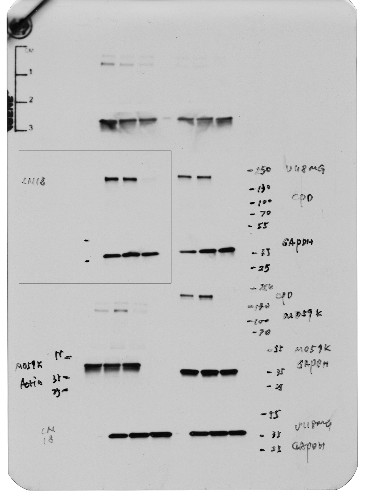


C D E


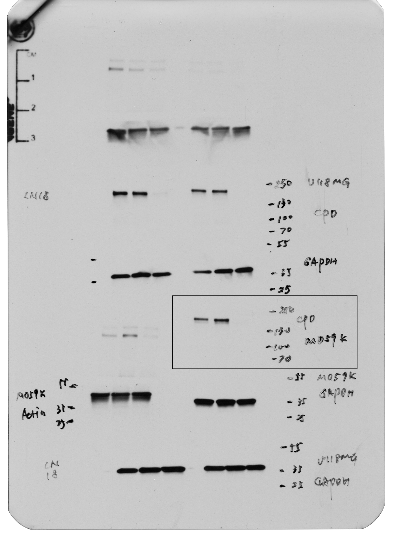






F G H


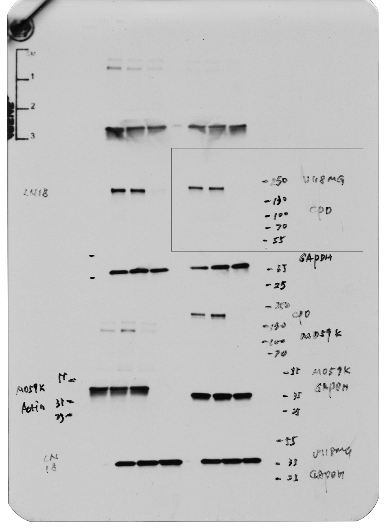






**Figure S4. Raw Western blots from Figure 5 in main text.** (A) CPD and GAPDH and (B) MET in LN18 cell line. (C) CPD, (D) GAPDH, and (E) MET in M059K cells. (F) CPD, (G) GAPDH, and (H) MET in U188MG cells.
