# Supplementary material for: Dynamic rewiring of biological activity across genotype and lineage revealed by context-dependent functional interactions
Source: Genome Biol. 2022 Jun 29;23:140. doi: 10.1186/s13059-022-02712-z (PMC9241233; doi:10.1186/s13059-022-02712-z)
Supplement: Supplementary file 5 — Additional file 5. Review history. [file 13059_2022_2712_MOESM5_ESM.docx]

Review History

**First round of review**

**Reviewer 1**

**Are you able to assess all statistics in the manuscript, including the appropriateness of statistical tests used?** Yes, and I have assessed the statistics in my report.

**Comments to author:**

The authors applied Pearson correlation analysis between gene essentiality and mutation status in cancer cell line data in this study. Their conditioned the correlation analysis on a third genetic status to identify context-specific functional interactions. In general, the manuscript is clearly written. Although previous studies, such as CEN-tools, introduced the concept of context-specific interaction, the current work extended it to gene mutational context. However, I have a few significant concerns.

Major concern 1, the webserver https://diffnet.hartlab.org is not working. When I log in to this server and tried to input gene and context, I did not see any response from the interactive network panel. Also, there is no help information available to guide users. A functional webserver for reviewers to evaluate is the precondition of considering such work for publication.

Major concern 2, lack of experiment validations on key conclusions. Besides known genetic interactions identified in this study, this study also reported a few more associations, such as the maturation of pro-receptors IGF1R and MET by proteases FURIN and CPD. Experimental validations will be essential to support these conclusions beyond reporting correlation values.

Major concern 3, lack of systematic computational validation. For computational frameworks, systematic performance validation is essential for readers to understand the expected accuracy. Although it is challenging to define an evaluation standard for context-dependent genetic interactions, the authors may consider defining such a "gold standard" focused on a few well-studied oncogenic pathways or through cross-validations between genetic screening datasets generated from different centers.

**Reviewer 2**

**Are you able to assess all statistics in the manuscript, including the appropriateness of statistical tests used?** Yes, and I have assessed the statistics in my report.

**Comments to author:**

Dynamic rewiring of biological activity across genotype and lineage revealed by context-dependent functional interactions.
Eiru Kim, Lance C. Novak, Veronica Gheorghe, Christopher A Bristow, Traver Hart

Summary: The authors devise an in silico framework for identifying cancer dependency relationships that change markedly based on tissue or genomic lesion variations. For each of 808 cell lines, log Bayes Factor (logBF) essentiality scores were computed via BAGEL2 based on reads from DepMap 20Q4. Over 36,000 potential "features" were developed based on cell line sequence, expression, and metadata obtained from CCLE. This list of 36,000 was then filtered down to 2,918 features. The logBF scores were binarized to facilitate penalized logistic regression. Only about a sixth (2,987 out of 18,094) of genes had binarized logBF vectors with between 1% and 80% "essential" values, and these were the only genes analyzed in the elastic net logistic regression, with a separate model fit for each gene. This resulted in (2,918 feature coefficients / model) x (2,987 models) > 8.7e6 feature coefficients across all models, very many of which will be shrunk to zero. Only coefficients exceeding 1.2 in absolute value were retained, and this list was found to span 393 of the 2,918 features tested. These 393 features were described as being "predictive of gene essentiality". For each of these 393 features in turn, a subset of cell lines exhibiting a given feature could be explicitly listed out. In order to identify genes that covaried in essentiality only in the context of a feature, the following steps were taken:

a. The Pearson correlation coefficient for the logBF values between all pairs of genes across all 808 cell lines was found (call this "PCC_all"). I could not discern whether this was between all pairs for 18,094 genes or only the 2,987 genes with models fit with the penalized elastic net -- did I miss this?

b. For each of the 393 features in turn, the cell lines exhibiting that feature's "context" were dropped and the Pearson correlation was computed on the remaining cell lines (so something lower than 808, but the number could vary across the 393 features). Call This number "PCC_~context_i" for i = 1, ..., 393.

c. Compute dPCC_i = PCC_all - PCC_~context_i for i = 1, ..., 393.

d. Statistical significance of the {dPCC_i} values was determined essentially by a Monte Carlo scheme, dropping random subsets of the correct numbers of cell lines for each feature, recomputing fake "dPCC_i" values, and comparing these to the values found by dropping the cell lines actually exhibiting the feature.


If the above is more or less correct, here are a few questions / suggestions:

1. Please comment on the advantage of (or at least preference for) binarizing the logBF values and doing logistic regression instead of just leaving them as continuous and doing an elastic net with continuous response, perhaps winsorizing in the case of extreme outliers. In the text (line 89), the authors say binarization was because penalized logistic regression "handles binary predictors, … gene essentiality is readily binarized…, and … the L1 component of the elastic net forces most coefficients to zero". The first and third points are also true of elastic nets with continuous response, while the second is not a reason that binarization is a good idea, just that it's feasible.

2. I do not see anywhere in the methods where the authors actually say that they are finding correlations of logBF factors (as opposed to, e.g., expression levels across cell lines or something else). I am surmising this based on poring over figure legends and Fig 2A axis labels. Did I miss this?

3. Similarly, I don't know whether the number of genes considered in the correlation was 18,094 or 2,987 or something else. I know I noted this above, but it will be important to clarify.

4. Did the authors consider or test out using Spearman or Kendall correlations in lieu of Pearson correlations? This would quite possibly dampen the range of differenced correlations, but would seem consistent with binarizing the logBF in the elastic net, and also provide some assurance that results were not driven by extreme logBF values concordant across two genes for a handful of cell lines.

5. I appreciate that the authors' code will be made available (in fact, I agree this is necessary), but do not think that readers should feel obliged to go pore over python scripts to try to figure out what the key details of the methods were, which seems to be recurring advice in the text. I think this is an interesting and promising piece of work, but frankly the methods section doesn't feel as if it was crafted extensively enough to help the reader understand things easily (e.g., it took quite a while for me to piece together the < 1 page description above). I encourage the authors to try rewriting -- perhaps allocating more words, which I know may be at a premium -- to make their interesting approach easier to follow.

6. Making the author's approach available through an online tool is a great idea and should make it more approachable for readers.

**Authors Response**

**Point-by-point responses to the reviewers’ comments:**

*Reviewer #1: The authors applied Pearson correlation analysis between gene essentiality and mutation status in cancer cell line data in this study. Their conditioned the correlation analysis on a third genetic status to identify context-specific functional interactions. In general, the manuscript is clearly written. Although previous studies, such as CEN-tools, introduced the concept of context-specific interaction, the current work extended it to gene mutational context. However, I have a few significant concerns.

Major concern 1, the webserver* [*https://diffnet.hartlab.org*](https://diffnet.hartlab.org/) *is not working. When I log in to this server and tried to input gene and context, I did not see any response from the interactive network panel. Also, there is no help information available to guide users. A functional webserver for reviewers to evaluate is the precondition of considering such work for publication.*

We apologize for having a site with such marginal functionality up during review. It was still in progress. We hope that your experience with the new and vastly improved diffnet will be better. We have added a brief paragraph describing the webstite:

**Diffnet: explore dynamic functional interactions**

A website is available at https://diffnet.hart-lab.org for interactive visualization of context-dependent functional interactions. Users can browse or search predefined sets of genetic lesions or tissue/tumor types and see the networks that are associated with differential coessentiality in that context as well as a table of network edges along with key parameters, including PCC, dPCC, and empirical P-values. Clicking any edge in the network view will display a scatterplot of logBF scores for the two connected genes across all cell lines in the DepMap data, annotated by whether they are in the selected context. A screenshot of the BRAF/RAF1 change in interaction in the BRAF_GOF context is shown in Supplementary Figure 2.

*Major concern 2, lack of experiment validations on key conclusions. Besides known genetic interactions identified in this study, this study also reported a few more associations, such as the maturation of pro-receptors IGF1R and MET by proteases FURIN and CPD. Experimental validations will be essential to support these conclusions beyond reporting correlation values.*

We have included an experimental validation of the role of CPD in posttranslational processing of the MET receptor in the text and in Figure 5. In glioma cell lines, knocking out CPD changes the ratio of proMET/MET by a factor of 3. We have updated the text as follows:

To test this prediction, we used a dual-guide CRISPR/enCas12a vector (see Methods) to knock out either *CPD* or negative control *ADH7* in glioblastoma cell lines and examined *MET* protein. We selected three brain cancer cell lines from PICKLES (Lenoir et al., 2018) where the *MET* receptor was expressed but not essential (LN18, U118MG, and M095K). As measured by Western blotting, under normal conditions MET is more abundant than its precursor proMET, indicating efficient posttranslational maturation of the polypeptide (**Figure 5E**). In contrast, in CPD^-/-^ cells, the ratio of proMET to MET is, on average, 3.6 times that of parental cells (**Figure 5F**; Supplementary Table 3). These observations are consistent with *CPD* playing an important role in maturation of the *MET* proreceptor in glioblastoma cells.

**Figure 5E-F**

We note that the role of FURIN and CPD in maturation of IGF1R was previously reported and cited in the text. (Alarcón et al., 1994, Han et al., 2020)

*Major concern 3, lack of systematic computational validation. For computational frameworks, systematic performance validation is essential for readers to understand the expected accuracy. Although it is challenging to define an evaluation standard for context-dependent genetic interactions, the authors may consider defining such a "gold standard" focused on a few well-studied oncogenic pathways or through cross-validations between genetic screening datasets generated from different centers.*

Herein lies a problem! We completely agree that systematic performance validation is important, when there is an appropriate gold standard. To be precise, however, we are not measuring genetic interactions - we are measuring coessentiality relationships, which generally imply functional interactions, and more importantly we are assigning a particular context to some functional interactions. It less a question of whether such functional interactions exist in the general sense (these are effectively scored and validated in the various approaches to coessentiality networks – Wang 2017, Rauscher 2018, Boyle 2018, Pan 2018, Kim 2019, Wainberg 2021), but whether we can identify context-specific functional interactions. However, there is no such reference set for background-specific interactions – this is a major part of the novelty of this work. We demonstrate in Figures 2, 3, and 4 examples where we know the biology behind the context specificity, and experimentally validate a prediction in Figure 5. We provide substantially more detail describing our statistical approach in the Method section (see below). And additionally we now provide in Supplementary Table 2 a list of top hits out of the (393 x 18k x 18k / 2 = ) ~64 billion gene/gene/context interactions that we tested, many of which pass the “eye test”.

*Reviewer #2: Dynamic rewiring of biological activity across genotype and lineage revealed by context-dependent functional interactions. Eiru Kim, Lance C. Novak, Veronica Gheorghe, Christopher A Bristow, Traver Hart*

*Summary: The authors devise an in silico framework for identifying cancer dependency relationships that change markedly based on tissue or genomic lesion variations. For each of 808 cell lines, log Bayes Factor (logBF) essentiality scores were computed via BAGEL2 based on reads from DepMap 20Q4.  Over 36,000 potential "features" were developed based on cell line sequence, expression, and metadata obtained from CCLE.  This list of 36,000 was then filtered down to 2,918 features.  The logBF scores were binarized to facilitate penalized logistic regression.  Only about a sixth (2,987 out of 18,094) of genes had binarized logBF vectors with between 1% and 80% "essential" values, and these were the only genes analyzed in the elastic net logistic regression, with a separate model fit for each gene.  This resulted in (2,918 feature coefficients / model) x (2,987 models) > 8.7e6 feature coefficients across all models, very many of which will be shrunk to zero.  Only coefficients exceeding 1.2 in absolute value were retained, and this list was found to span 393 of the 2,918 features tested.  These 393 features were described as being "predictive of gene essentiality". For each of these 393 features in turn, a subset of cell lines exhibiting a given feature could be explicitly listed out.  In order to identify genes that covaried in essentiality only in the context of a feature, the following steps were taken:*

*a. The Pearson correlation coefficient for the logBF values between all pairs of genes across all 808 cell lines was found (call this "PCC_all"). I could not discern whether this was between all pairs for 18,094 genes or only the 2,987 genes with models fit with the penalized elastic net -- did I miss this?*

*b. For each of the 393 features in turn, the cell lines exhibiting that feature's "context" were dropped and the Pearson correlation was computed on the remaining cell lines (so something lower than 808, but the number could vary across the 393 features).  Call This number "PCC_~context_i" for i = 1, ..., 393.*

*c. Compute dPCC_i = PCC_all - PCC_~context_i for i = 1, ..., 393.*

*d. Statistical significance of the {dPCC_i} values was determined essentially by a Monte Carlo scheme, dropping random subsets of the correct numbers of cell lines for each feature, recomputing fake "dPCC_i" values, and comparing these to the values found by dropping the cell lines actually exhibiting the feature.*

*If the above is more or less correct, here are a few questions / suggestions:*

We thank the reviewer for taking the time to decipher our process. We have substantially revised the methods section to better describe the process. The reviewer’s description of the regression model is accurate, though we note that only one expression-based feature is included (CDH1/VIM ratio). For the next step, all the PCC calculations are performed for all pairs of all 18k genes.

*1. Please comment on the advantage of (or at least preference for) binarizing the logBF values and doing logistic regression instead of just leaving them as continuous and doing an elastic net with continuous response, perhaps winsorizing in the case of extreme outliers.  In the text (line 89), the authors say binarization was because penalized logistic regression "handles binary predictors, … gene essentiality is readily binarized…, and … the L1 component of the elastic net forces most coefficients to zero". The first and third points are also true of elastic nets with continuous response, while the second is not a reason that binarization is a good idea, just that it's feasible.*

As mentioned above, we have added considerable detail to our methods section, including this paragraph to address the reviewer’s important question:

Gene essentiality was binarized for use as a response variable. Genes with missing values in the Bayes Factor table (n=17) were removed from the dataset. Then, for each cell line, genes with BF>=10 were classified as essential and all others nonessential, for a set of 18,094 genes in 808 cell lines. We used a binary rather than continuous response variable because, in this study, we seek to identify predictors of whether a gene does or doesn’t have a fitness defect, not to predict variation in that defect. Moreover, while the magnitude of positive log BF may be a proxy for severity of fitness defect, the magnitude of negative log BF is usually not (though see (Lenoir et al., 2021) for exceptions). Bayes Factor combines statistical confidence and effect size into a single output, and the dynamic range of BF scores from each screen is highly dependent on that screen’s quality (Kim and Hart, 2021; Wang et al., 2020). Bayes Factors are not directly comparable across screens without normalization (see Global normalization, below). See notebook “step02-remove_duplicates” for details on this processing step.

Importantly, we use this regression step solely as a way to define the universe of contexts against which we test for background-specific coessentiality. While this matrix of regression coefficients might be interesting to the broader research community (we include it as a supplementary table), it is very much meant to be an intermediate component of the pipeline, not a standalone result or as a proposed “improvement” over Lord et al or Behan et al.

*2. I do not see anywhere in the methods where the authors actually say that they are finding correlations of logBF factors (as opposed to, e.g., expression levels across cell lines or something else).  I am surmising this based on poring over figure legends and Fig 2A axis labels.  Did I miss this?*

No, we just failed to address it. Again, in our more detailed methods:

**Measurement of emergent network rewiring in CRISPR dataset**

*Global normalization and correlation*

To calculate the baseline coessentiality network, we first quantile normalized the log BF table of 18,094 genes with no missing data x 808 cell lines. Quantile normalization was performed without a reference distribution, setting each gene to its rank mean; e.g. the top-ranked gene in each sample was assigned a value equal to the mean BF of all top-ranked genes in 808 samples. The global Pearson correlation network of all gene pairs was then calculated using the Python numpy function corrcoef, resulting in an 18k x 18k matrix of Pearson correlation coefficients hereafter referred to as PCC_all_.

*Leave-one-out test*

To test for dynamic functional network rewiring, we measured the differential Pearson correlation coefficient (dPCC) between the PCC_all_ global network and the network derived from a subset of cell lines that exclude a given context (PCC_~context_). The formula of dPCC for each gene pair is therefore:

$$dPCC={{PCC}_{all}- PCC}_{\sim context}$$

Where PCC_~context_ is an 18k x 18k matrix of Pearson correlation coefficients derived from the [ 18k x (808-*n*) ] matrix of quantile normalized BFs, and *n* is the number of cell lines associated with the ‘context’. Positive dPCC represents a **gain of interaction (GOI)** because the higher PCC_all_ depends on the presence of cells with the feature of interest, and negative dPCC represents **loss of interaction (LOI)** because excluding cells associated with the feature of interest increases the PCC.

We evaluated the statistical significance of the observed dPCC (dPCC_obs_) for each gene pair/feature combination empirically. Briefly, we determined *n*, the number of cell lines associated with the feature. Then we randomly removed *n* cell lines from the set of all 808 cell lines and calculated the all-by-all PCC matrix for all pairs of genes, PCC_rand_. For each gene pair, we measured dPCC_rand_ = PCC_all_ – PCC_rand_. We repeated this process 1000x and compared the observed dPCC_obs_ to the bootstrapped distribution of dPCC_rand_ to calculate a two-sided empirical P-value that dPCC_obs_ is higher or lower than random expectation. In general, our sampled null distributions of dPCC_rand_ are not well estimated by classical distributions, and our estimate of P-value is therefore limited to a discrete distribution in steps of 0.001. We repeated this bootstrap resampling to generate null distributions of dPCC_rand_ for every gene pair for each of the 393 contexts identified by the logistic regression model (a computationally expensive process). See “calc_diff_coess_pval.py” for code and details.

*3. Similarly, I don't know whether the number of genes considered in the correlation was 18,094 or 2,987 or something else.  I know I noted this above, but it will be important to clarify.*

Hopefully we have clarified it in the above excerpt from Methods.

*4. Did the authors consider or test out using Spearman or Kendall correlations in lieu of Pearson correlations?  This would quite possibly dampen the range of differenced correlations, but would seem consistent with binarizing the logBF in the elastic net, and also provide some assurance that results were not driven by extreme logBF values concordant across two genes for a handful of cell lines.*

The reviewer raises some very important points here that we needed to explicitly address in the text. In short, we _rely_ on the Pearson’s sensitivity to extreme logBF values. Again, from Methods:

It is important to note that this empirical approach does not rely the robust measurement of Pearson correlation – in fact, the approach described here exploits the Pearson’s lack of robustness in the presence of outliers. When the outliers are functionally coherent in some aspect (e.g. breast cancer cells in Figure 2G, glioma cells in Figure 5D), their removal in the leave-one-out test and the resulting major change in PCC is precisely the result of the Pearson’s sensitivity to outliers, and would likely not be detectable with nonparametric alternatives such as Spearman’s rank correlation that are designed to be insensitive to such changes. Of further importance is that we do not evaluate the statistical significance of any Pearson correlation, in large part because of these concerns. Our only test statistic is dPCC, measured against null distributions that we sample empirically.

*5. I appreciate that the authors' code will be made available (in fact, I agree this is necessary), but do not think that readers should feel obliged to go pore over python scripts to try to figure out what the key details of the methods were, which seems to be recurring advice in the text.  I think this is an interesting and promising piece of work, but frankly the methods section doesn't feel as if it was crafted extensively enough to help the reader understand things easily (e.g., it took quite a while for me to piece together the < 1 page description above). I encourage the authors to try rewriting -- perhaps allocating more words, which I know may be at a premium -- to make their interesting approach easier to follow.*

Many more words have been allocated!

*6. Making the author's approach available through an online tool is a great idea and should make it more approachable for readers.*

The approach itself is fairly resource intensive – approx. 250 CPU-days to calculate the null distributions of every dPCC/context. “Interesting” results are quite rare (less than 3,000 out of billions tested) and it is not obvious how to estimate a false discovery rate. However, such results as we have are available to browse through our improved web interface at diffnet.hart-lab.org and for download as Supplementary Table 2. Of course all our code for the pipeline and the website itself are freely available.

**Second round of review**

**Reviewer 1**

The authors have addressed my previous concerns. I think the current form of this manuscript is suitable for publication.

**Reviewer 2**

Thank you to the authors for considering my principal concerns and addressing them thoroughly. I also revisited the website (I also had found it lacking, though it was the other reviewer you spoke more to this) and it appears to work now.

The methods section is much more comprehensible than in the first version and I expect that readers will be able to follow what was done without much trouble. This broadly covers much of my concern from the initial round of review. The addition of a small biological confirmation study knocking down CPD (or ADH7 as a negative control) and evaluating its effects on the relative amounts of proMET vs. MET in glioma cell lines (Figure 5, and the text) is a nice addition that should provide readers with some confidence regarding other context-dependent functional interactions.
